# COVID-19 therapeutics: Challenges and directions for the future

**DOI:** 10.1073/pnas.2119893119

**Published:** 2022-04-06

**Authors:** Philip C. Robinson, David F. L. Liew, Helen L. Tanner, John R. Grainger, Raymond A. Dwek, Ronald B. Reisler, Lawrence Steinman, Marc Feldmann, Ling-Pei Ho, Tracy Hussell, Paul Moss, Duncan Richards, Nicole Zitzmann

**Affiliations:** ^a^Faculty of Medicine, University of Queensland School of Clinical Medicine, Herson, QLD 4006, Australia;; ^b^Metro North Hospital & Health Service, Royal Brisbane & Women’s Hospital, Herston, QLD 4029, Australia;; ^c^Department of Medicine, University of Melbourne, Melbourne, VIC 3010, Australia;; ^d^Department of Rheumatology, Austin Health, Melbourne, VIC 3084, Australia;; ^e^Department of Clinical Pharmacology and Therapeutics, Austin Health, Melbourne, VIC 3084, Australia;; ^f^Lydia Becker Institute of Immunology and Inflammation, The University of Manchester, Manchester M13 9PL, United Kingdom;; ^g^Oxford Glycobiology Institute, Department of Biochemistry, University of Oxford, Oxford OX1 2JD, United Kingdom;; ^h^Medical Division, Davis Defense Group, Stafford, VA 22554;; ^i^Department of Neurology and Neurological Sciences, Stanford University, Stanford, CA 94305;; ^j^Department of Pediatrics, Stanford University, Stanford, CA 94305;; ^k^Kennedy Institute of Rheumatology, Nuffield Department of Orthopaedics, Rheumatology, and Musculoskeletal Sciences, Botnar Research Centre, University of Oxford, Oxford OX3 7LD, United Kingdom;; ^l^Medical Research Council Human Immunology Unit, University of Oxford, Oxford OX3 7LD, United Kingdom;; ^m^College of Medical and Dental Sciences, University of Birmingham, Birmingham B15 2TT, United Kingdom;; ^n^Oxford Clinical Trials Research Unit, Nuffield Department of Orthopaedics, Rheumatology, and Musculoskeletal Sciences, Botnar Research Centre, University of Oxford, Oxford OX3 7LD, United Kingdom

**Keywords:** immunology, virology, epidemiology

## Abstract

The emergence of SARS-CoV-2 triggering the COVID-19 pandemic ranks as arguably the greatest medical emergency of the last century. COVID-19 has highlighted health disparities both within and between countries and will leave a lasting impact on global society. Nonetheless, substantial investment in life sciences over recent decades has facilitated a rapid scientific response with innovations in viral characterization, testing, and sequencing. Perhaps most remarkably, this permitted the development of highly effective vaccines, which are being distributed globally at unprecedented speed. In contrast, drug treatments for the established disease have delivered limited benefits so far. Innovative and rapid approaches in the design and execution of large-scale clinical trials and repurposing of existing drugs have saved many lives; however, many more remain at risk. In this review we describe challenges and unmet needs, discuss existing therapeutics, and address future opportunities. Consideration is given to factors that have hindered drug development in order to support planning for the next pandemic challenge and to allow rapid and cost-effective development of new therapeutics with equitable delivery.

From the outset, COVID-19 has been an infection with significant mortality. As the pandemic swept across the world, we urgently needed to determine effective and optimal therapies for patients at each stage of disease. However, as a novel virus, there was very little information to guide choice of therapy and considerable debate about which agents to include in clinical trials.

The enormity of the pandemic has led to a welcome focus on collecting only that information which informs decision making in clinical trials, but also to a plethora of trials that did not collect information on enough patients to be truly informative. Therapeutic strategy has been lacking; some potential therapies have been tested multiple times (e.g., >150 hydroxychloroquine trials), while other promising approaches have yet to be tested at scale (e.g., anti–GM-CSF [granulocyte-macrophage colony-stimulating factor], anti-TNF [tumor necrosis factor]). Future efforts need to be more coordinated without stifling innovation.

Ideal COVID-19 treatments are inexpensive, with a good safety profile, and prevent clinical deterioration and hospitalization. Currently, the only therapies with published evidence of benefits early in the course of infection are inhaled interferon (IFN), small molecules, and monoclonal antibodies (mAbs). All are expensive, with limited availability and, hence, are impractical for the majority of the world’s population. We also have a limited understanding of on whom to focus early treatment. Age, obesity, diabetes, hypertension, and immune compromise increase the likelihood of clinical deterioration, but we need biomarkers to better predict progression. For patients in hospitals, there are approved therapies that are effective in a subset of severe cases—namely dexamethasone, tocilizumab, and remdesivir—but again the costs of the latter two are prohibitive for most countries. It is clear that we need cost-effective drugs that prevent disease progression and are easy to administer in all countries, including low- and middle-income countries.

Here we review all aspects of COVID-19 therapeutics. We explore challenges that have stymied COVID-19 drug development and highlight important unmet needs. We examine key therapeutic classes and identify promising novel approaches for the current and future pandemics. Therapeutic strategy is always controversial and we recognize that the opinions expressed here will not meet with universal agreement, but we have endeavored to be inclusive in our assessment.

## Challenges and Unmet Needs

### Clinical Trials during a Pandemic.

Pandemics stress the clinical trial process and conventional trial set-up processes are too slow for a rapidly spreading pathogen. Prepandemic clinical trial networks demonstrated their value by enabling rapid recruitment, such as with Randomized, Embedded, Multifactorial Adaptive Platform Trial for Community-Acquired Pneumonia (REMAP-CAP) (1) in intensive care unit (ICU) patients. In contrast, for non-ICU hospitalized or primary care patients, there was no established framework. In these key populations, early drug development was characterized by small, underpowered clinical studies of repurposed drugs, often with weak methodology; even with meta-analysis, the information was not of sufficient quality to inform clinical practice. A comprehensive clinical trial system is required for a pandemic response, to ensure a sustained and effective pipeline of drug development across clinical trial phases. Clinical trial legislation requires that a trial is either open and recruiting or that it is not, and therefore it is currently logistically and financially challenging to maintain large clinical trial networks outside of pandemic settings. Some of the most nimble and responsive studies (International Severe Acute Respiratory and Emerging Infection Consortium and REMAP-CAP) have been those created to address epidemics and seasonal outbreaks that were consequently able to pivot rapidly to address the pandemic. It is anticipated that some of the platform studies will evolve to address common infections, such as influenza. While this will require a significant long-term resource commitment and legislative support, it offers the exciting possibility of rapidly generating definitive therapeutic guidance in a way not previously possible. The greatest advances have come from using existing tools better (e.g., data linkage, adaptive platforms) rather than inventing new ones. Interestingly, we have yet to see novel insights from pooling datasets.

The most important innovation came with large, pragmatic outcome adaptive platform studies: RECOVERY, SOLIDARITY, PRINCIPLE, and ACTIV (2–5). The largest, RECOVERY, has provided practice-changing evidence of benefits in hospitalized patients for three therapies (dexamethasone, tocilizumab, and the neutralizing monoclonal antibody combination casirivimab/imdevimab) and ruled out significant benefits for six therapies (aspirin, azithromycin, colchicine, convalescent plasma, lopinavir/ritonavir, and hydroxychloroquine). Adaptive platform studies represent a step forward for demonstrating clinical effectiveness that previously was based on manufacturer-sponsored phase 3/4 studies. Their key features are a clear focus on hard endpoints with an economy of data collection, commonly using routinely collected data. They are adaptive, allowing ineffective interventions to be dropped and new candidates brought in.

Rapid delivery of platform studies required an unprecedented level of cooperation between trial teams, drug manufacturers, regulators, and health system managers. This unified “top-down” approach is necessary to prioritize candidates for testing; large numbers are needed for definitive data, so the number of candidates that can be tested is limited. It has taken longer to formalize effective oversight processes than to start the studies themselves.

Without question, the seemingly rapid development of COVID-19 vaccines has been a cornerstone of the COVID-19 response and might raise comparisons to the relative speed of drug development for COVID-19. Such comparisons highlight the reason for the rapid appearance of multiple COVID-19 vaccines: decades of investment in preparatory work, refining both science and application via strong collaborative efforts, leading to technology that was ready to be repurposed when needed. While the process of drug development is arguably subject to greater variability, the greatest successes in COVID-19 drug development so far have come where strong scientific investigation was in place many years prior to the emergence of the pathogen. Readiness for the next pandemic requires similar investment in preparatory work, with strong collaboration and sensible oversight.

Outcome measures affect the speed of drug development. Phase 3 studies work best when fed preliminary data from fast, efficient phase 2 studies driven by robust surrogate markers for the clinical endpoint, namely death. The absence of surrogate markers in COVID-19 has meant that most phase 2 studies have employed clinical outcomes (World Health Organization [WHO] ordinal scale). Such tools require large numbers, leading to long recruitment times. As a result, data have not been available in a timely manner to inform drug selection for phase 3 platform trials. The phase 2 CATALYST study was one of very few to employ a Bayesian rather than frequentist model-based design using a biological surrogate (C-reactive protein, CRP) (6). This approach showed a clear difference between namilumab (antigranulocyte-macrophage colony-stimulating factor, anti–GM-CSF) and infliximab (antitumor necrosis factor, anti-TNF) based on small numbers of subjects (30 to 60 per arm), although neither agent has completed phase 3 testing to validate the predictive value of this surrogate.

It is clear we need to establish reliable surrogates for death in order to expedite phase 2 trials. This will become increasingly more important as combination therapies become the norm and regulators insist that combination therapy includes treatments that are difficult to access, such as mAbs.

### Early Treatment and Disease Stage.

The clinical course of COVID-19 is heterogeneous and may be prolonged. A major challenge with drug development has been tailoring therapies to the stage of disease. Most clinical trial data currently apply to hospitalized patients. There is limited insight into effective treatments for the early stages or in the posthospital or long-COVID setting.

**Fig. 1.**
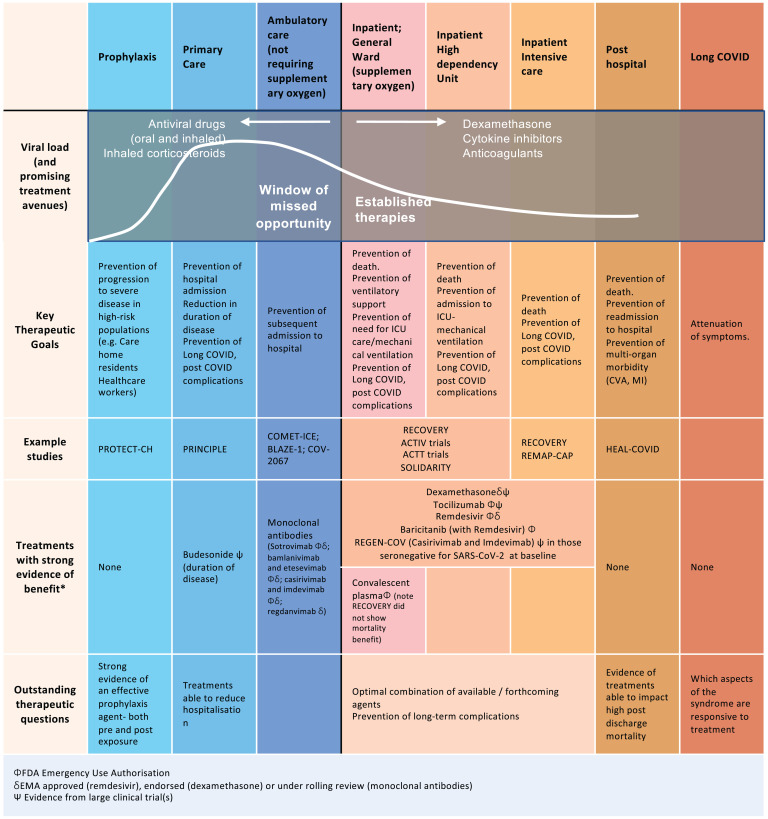
Established therapies and future opportunities for intervention early in disease. Following infection with SARS-CoV-2, there are specific time points in the disease trajectory where different therapies could be optimally administered. At this time, the majority of treatments have been targeted during hospitalization and particularly at late stages of acute disease during ICU admission. Additionally, many of the therapies trialed are antibody therapies and are cost prohibitive, especially in low- to middle-income countries. There is currently a window of missed opportunity early in disease to reduce progression to hospitalization.

There is a window of missed opportunity: Early treatment, by reducing progression to hospitalization, might substantially reduce long-term morbidity and mortality and reduce the burden on healthcare resources (Fig. 1). For an early-intervention strategy to work, we need to accurately identify patients at greatest risk of clinical deterioration. Demographic factors help, as well as laboratory measures like elevation of CRP (7–9). Deep phenotyping studies, incorporating both clinical features and immunological biomarkers (10) and utilizing machine-learning techniques, are likely to deliver a more sophisticated understanding necessary to identify best targets for early therapy.

### Long-COVID.

An important emerging issue is the symptom complex long-COVID. Prevalence appears high in both hospitalized patients and those with mild/moderate disease (11–13). Evidence suggests that long-COVID is more severe than other postviral syndromes. Patients admitted to hospital with COVID-19 had significantly higher rates of death, cardiovascular disease, neurological and mental health disorders, fatigue, and coagulation disorders than those admitted with influenza (14).

The mechanism of long-COVID is not well understood. Theories include long-term cellular damage caused by the SARS-CoV2 virus during acute infection, SARS-CoV-2 persistence, postacute illness, and the development of autoimmunity (14, 15). Changes to social circumstances, including job loss and social isolation, may also negatively impact the clinical course. Without a clear understanding of the pathogenesis of long-COVID, the development of effective therapy is challenging.

Encouragingly, studies are shedding light on the pathogenesis of the most common complaints in long-COVID, namely pulmonary and neurologic symptoms (14, 16). The highly sensitive hyperpolarized Xenon magnetic resonance imaging method of visualizing microstructure abnormalities in the lung shows persistent anomalies at the alveolar-endothelial-epithelial junction (17). Neuropathology studies, mainly from autopsies, show abnormalities similar to dementia, schizophrenia, and depression (18). Brain specimens show evidence of widespread inflammatory changes, including lymphoid infiltration and microglial activation (19). Coagulation abnormalities are also common (20, 21), with brain infarcts seen in about 20% of individuals and microthrombi and hemorrhages seen in 15% of individuals. These abnormalities might help to explain the symptoms of “brain fog” and fatigue in those with long-COVID.

Therapeutics currently under investigation for long-COVID target different proposed mechanisms for pathogenesis. Strategies include antifibrotic medication due to the observation of fibrotic lung changes, anticoagulants, statins for their anti-inflammatory properties, and dedicated long-COVID rehabilitation centers (22–24). Observational data also suggests improvement in long-COVID symptoms postvaccination, the theory being that vaccination induces elimination of residual SARS-CoV-2 virus (25).

### Vulnerable Populations.

Low- to middle-income countries will likely bear the greatest disease burden from COVID-19 due to delayed access to effective vaccination and the consequences of structural inequity, such as overcrowding. These countries need affordable, easily administered COVID-19 therapies, such as temperature-stable oral medicines or inhaled therapeutics. Robust and accountable collective purchasing and distribution systems will need to stand independently of national interest. Collective drug purchasing, driven by the WHO and nongovernment initiatives, like the Global Fund and the Gates Foundation, has proven effective in ongoing challenges, such as HIV/AIDS. A similar approach in COVID-19 is necessary (26), accompanied by preventative strategies and culturally appropriate community engagement, particularly as COVID-19 will likely persist as a global threat until all countries can sustainably access treatment.

Even with widespread COVID-19 vaccination uptake, there will always be people with poor vaccination response. Immunomodulatory medications like glucocorticoids and rituximab lead to reduced response to SARS-CoV-2 vaccines (27), and it is possible that other immune-modifying therapies cause a similarly reduced response. Without herd immunity, these patients will remain vulnerable to severe infection. Huge resources have appropriately been allocated to the development and distribution of effective vaccines, and this remains one of the great successes of the pandemic. Despite this, effective and cheap therapeutics are critical given viral genome evolution, particularly for those who do not sufficiently respond to vaccines, for those who decline to receive vaccines, for those yet to be able to access vaccines on scale, and as a back-up should variants of concern evade vaccine-induced immunity. While vaccine development has been rightfully relatively prioritized to this point, the efforts of the medical and scientific community will only be complete with a renewed focus on therapeutics.

This focus on therapeutics will be particularly important for certain populations. Patients on B cell-depleting therapies, used widely as a treatment in hematological malignancy, rheumatology, clinical immunology, and neurology, are particularly vulnerable. B cell-depleting therapy has been identified as a significant inhibitor of COVID-19 vaccine serological responses, but our understanding of cell-mediated immunity is more primitive (28). For these patients, and for those with inherited or acquired immunodeficiency, preexposure prophylaxis with long-acting neutralizing monoclonal antibodies clearly has a role, given that time is critically of essence for antibody generation if one wants to survive infection (29). Given that this approach does not rectify the underlying inadequate host immune response, efforts should also be focused on how we can prime these abnormal immune systems in focal ways to generate some immunity. This will be both a scientific and a logistical challenge, given the relative few who stand to benefit, but success in these efforts will likely have huge ramifications for those who are born with immune deficiencies or inherit them from treating cancer and autoimmune disease, and the broader measures required to protect them.

## The COVID-19 Therapeutic Landscape

At different stages of COVID-19, pathology and relevant targets differ (Fig. 1). In the early stages, antivirals are key, compared to later in the disease where inflammation and its consequences are largely culpable. Regrettably, the important timing of therapy administration within relevant windows of opportunity remains inexact. In fact, while some overall success has been achieved in all therapeutic classes, the choice and best use of agents is evolving rapidly.

### Antivirals.

Antivirals justifiably remain the focus for treatment in early COVID-19. Two main approaches exist: direct acting antivirals (DAAs) that target viral proteins, and less developed host-targeting agents (HTAs) that inhibit the human host cells that viruses need for replication and spread.

The most developed DAA candidates for COVID-19 are repurposed drugs. Remdesivir, an intravenously administered polymerase inhibitor developed for hepatitis C is one of the only currently approved antiviral option for COVID-19, but with limited benefit: reducing median recovery time (10 vs. 15 d) but not mortality (hazard ratio for death 0.73; 95%CI, 0.52 to 1.03) in the ACTT-1 trial (5) (*n* = 1,062). Subgroup analysis suggested greater benefit with early administration. In contrast, the SOLIDARITY trial (2) (*n* = 11,330) found no survival benefit with remdesivir (rate ratio for death of 0.95, 95%CI 0.81 to 1.11), and consequently the WHO issued a conditional recommendation against its use in hospitalized patients. While inducible resistance to remdesivir has been reported (30), variants do not appear to lead to preexisting resistance to remdesivir (31, 32). Another repurposed polymerase inhibitor is molnupiravir, originally developed for Venezuelan equine encephalitis and influenza. It is a prodrug that undergoes rapid conversion to the active nucleoside triphosphate, which is a competitive substrate for virally encoded RNA-dependent RNA polymerase. On incorporation into the nascent chain RNA, it increases mutational frequency and hence viral “error catastrophe.” Promising data from an interim analysis of the phase 3 study showing an approximate 50% reduction in hospitalization or death has triggered emergency use authorization in the United States and approval in the United Kingdom (33). As the experience with influenza and oseltamivir has shown (34), there is potentially a narrow window where DAA can work, and this may be why remdesivir has not shown better efficacy in inpatient trials.

Drugs that specifically target SARS-CoV-2 will likely have greater COVID-19 DAA potency than repurposed therapeutics; their development requires detailed structural knowledge of viral proteins. For SARS-CoV-2, the first relevant protein to be understood was the main protease, 3CLpro, as it is closely structurally related to the SARS-CoV-1 main protease; this understanding has accelerated development of protease inhibitors targeting it. PF-07321332/nirmatrelvir blocks the SARS-CoV-2/3CL protease and is delivered in combination with ritonavir. Ritonavir inhibits CYP3A4, prolonging exposure to PF-07321332/nirmatrelvir. A scheduled interim analysis showed an 89% reduction in risk of COVID-19–related hospitalization or death from any cause compared to placebo in patients treated within 3 d of symptom onset (NCT04960202). In both cases treatment needs to be started early in the illness (<5 d from symptoms). Their role in asymptomatic disease is unclear. The final analysis of the phase 3 studies and adverse effect profile is eagerly awaited and further assessment of effectiveness is likely to be required.

Other DAA targeting other SARS-CoV-2 proteins are under development, including those targeting polymerase, papain-like protease, helicase, and viral replication transcription complexes (RTC) responsible for viral RNA synthesis, proofreading, and 5′-capping. RTC are critically dependent on the interaction of at least nine different viral proteins, and these interfaces present additional targets. Other targets are constrained secondary RNA structures in the 5′ untranslated region, the frame-shift motif of the SARS-CoV-2 genome, the host protease TMPRSS2 utilized for viral entry, dihydroorotate dehydrogenase, and SIRT2 (35).

An interesting potential antiviral agent is a soluble recombinant form of the ACE2 receptor that prevents binding of the viral spike protein to cell-bound ACE2, reducing SARS-CoV-2 load in vivo (36) and potentially overcoming spike protein variant escape. Remdesivir and recombinant soluble ACE2 target different modalities of the SARS-CoV-2 life cycle, and in vitro the combination lengthened therapeutic windows against SARS-CoV-2 (37).

Challenges exist in prioritizing candidates for development. Drugs are often selected on the basis of activity in cell culture systems (sarscov2.assaytracker.net) with limited consistency, often due to the different cell types used for screening. Small-animal disease models poorly mirror some aspects of human disease, such as extrapulmonary manifestations. Human testing is vital but establishing early clinical proof-of-concept is challenging, partially due to a lack of defined standardized antiviral clinical trial endpoints.

The ideal antiviral agent in the current pandemic would be potently active against both current and future variants, with a good safety profile. One approach against variants, which would also assist in future pandemics, is to develop drugs with broad potential against viral families with pandemic potential: not only other coronaviruses, but also orthomyxoviruses, henipaviruses, filoviruses, and others. One option is HTAs that inhibit human host cell proteins responsible for viral replication and spread. The host is constant and less likely to drive escape variants, making HTAs truly broad-spectrum.

Historically, investment in HTA development has been poor, resulting in few options in use. However, the potential of broad-spectrum antivirals has recently been recognized by the National Institute of Allergy and Infectious Diseases and the Coalition for Epidemic Preparedness Innovation, but finding agents to directly act against common targets has proven elusive as viruses within and across families are extremely divergent. Developing HTAs takes time, as does DAA development. However, HTA-development does not require detailed structural knowledge of viral proteins and agents could therefore be developed in between outbreaks and used at the outset of the next pandemic. Safety concerns are often raised with HTAs, given they inhibit host targets; however, nearly all our therapeutics target the host, and all therapies, even DAAs, have the potential for toxicity, so this should not deter us from developing HTAs.

One promising target for HTAs is protein glycosylation, in particular enzymes involved in glycan-mediated endoplasmic reticulum quality control (ERQC) of viral glycoprotein folding (38). Iminosugars, which interfere with ERQC enzymes and have activity against SARS-CoV-2 (39), are orally available small-molecule drugs that are cheap to produce. Miglustat, an off-patent approved repurposable iminosugar, showed antiviral effects against SARS-CoV-2 in various cellular screens (39) and is due for testing in the proposed PLAT-COV platform trial. MON-DNJ, another iminosugar with phase 1 trial data, is active against SARS-CoV-2 in a cellular screen using human lung epithelial cells and reduces mortality in mice infected with influenza and dengue virus (40). Strategies against future viral threats might utilize such cost-effective orally available broad-spectrum antivirals. Soon after emergence of the virus, they might be deployed to reduce epidemic potential, and once virus-specific DAAs are developed, they could be included in more effective combination therapies that might also delay the emergence of variants of concern. Developing a suite of HTAs is a high priority for this pandemic and as insurance for the future.

### SARS-CoV-2–Targeting Neutralizing mAbs.

SARS-CoV-2 mAbs bind to the receptor binding domain of the SARS-CoV-2 spike glycoprotein, preventing viral entry into host cells. The fine specificity of mAbs limits their potential “off-target” toxicity but also makes them vulnerable to emergence of viral variants. In patients with mild-to-moderate COVID-19, bamlanivimab-etesevimab significantly reduced the composite outcome of hospitalization, emergency department visit, or death (7.2% vs. 2.3%) (41). The emergency use authorization for using bamlanivimab alone was revoked less than 6 mo after approval due to the increasing prevalence of viral variants, resulting in an increased risk of treatment failure (42, 43). Sotrovimab, derived from a survivor of SARS-CoV-2 infection, targets a conserved epitope in the receptor binding domain away from the ACE2 binding site and may therefore maintain effectiveness with viral variants (44), but this is not guaranteed.

Recently, mAbs were found to have increased efficacy in patients who were seronegative for SARS-CoV-2 antibodies (43, 45, 46). Some data have suggested that the use of mAbs in hospitalized patients with COVID-19 pneumonia and decreased oxygen saturation is associated with worse outcomes in patients with existing antibodies to SARS-CoV-2 (47). More recently in RECOVERY, hospitalized SARS-CoV-2 seronegative patients treated with mAbs had a reduced 28-d mortality, while seropositive patients had no benefit. Early antibody levels in COVID-19 may predict outcome in hospitalized patients (48). The intravenous route of administration presents a barrier to access for many patients. While the casirivimab-imdevimab emergency use authorization was recently modified to allow for four subcutaneous injections when intravenous administration is not feasible, this approach is currently unproven. Casirivimab-imdevimab has now also been shown to be effective at reducing symptomatic infection when used as postexposure prophylaxis (1.5% vs. 7.8%). There is also discussion about the use of mAbs as preexposure prophylaxis. Comprehensive data on this approach is still awaited, however, the combined therapeutic tixagevimab-cilgavimab has been given emergency use authorization for preexposure prophylaxis (49, 50). High cost, limited availability, and logistical challenges have limited the public health impact of mAbs.

### Anti-inflammatory Therapies.

In common with other respiratory viruses, SARS-CoV-2 induces a “cytokine storm” or “cytokine release syndrome” in the most severely affected patients. Medicines directed at suppressing inflammation play an important role in therapy.

Experience from SARS-CoV-1 and Middle East respiratory syndrome suggested that glucocorticoids did not confer benefit in inflammatory lung disease (51). However, the RECOVERY trial (3) and subsequently other randomized trials demonstrated reduced mortality with corticosteroids in COVID-19 (52–54). The benefit in the RECOVERY trial (*n* = 6,425) was seen in those who were mechanically ventilated or receiving supplemental oxygen. Overall, 22.9% patients in the dexamethasone group and 25.7% in the usual care group died within 28 d of randomization (age-adjusted rate ratio, 0.83; 95%CI 0.75 to 0.93). Subanalyses showed this difference was most marked among patients receiving invasive mechanical ventilation (29.3% vs. 41.4%) and those receiving oxygen without invasive mechanical ventilation (23.3% vs. 26.2%). Current recommendations both in the United Kingdom and from the NIH in the United States are for all patients requiring hospitalization for supplemental oxygen treatment to receive 6 mg of dexamethasone per day for 10 d. Corticosteroids can be given orally or intravenously and are not expensive or cumbersome to distribute, making them a relatively accessible therapy.

**Fig. 2.**
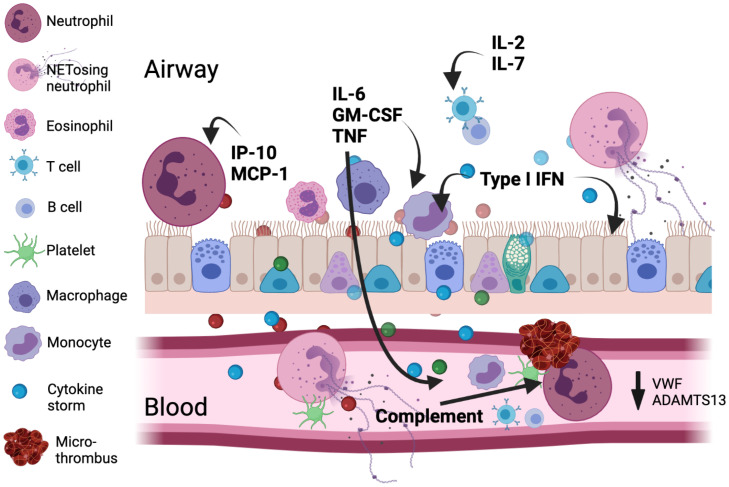
Major immunologic and coagulatory factors implicated in COVID-19 pathology. Viral infection leads to type I IFN, inflammatory mediator, and alarmin release from the respiratory epithelium/endothelium and resident immune cells setting-up a chemotactic gradient pulling cells from circulation into the lung. An emergency myelopoietic state occurs and neutrophils and monocytes display abnormalities in the blood in this state of high inflammation. Lymphocytes concurrently become depleted in circulation. Activated monocytes and macrophages can be an important source of cytokines, including IL-6. Enhanced by complement, clusters of neutrophils and activated platelets occur and neutrophil NETosis in blood and tissue directly augments thrombosis by supporting platelet activation. Size for each cell indicates its relative abundance in each compartment.

Many inflammatory mediators are induced in COVID-19, including interleukin (IL)-1, IL-2, IL-6, IL-7, GM-CSF, IFN-α–inducible protein 10 (IP-10), monocyte chemoattractant protein 1a (MCP1), and TNF (55–58). The excessive cytokine production drives abnormalities in many different cell types, including neutrophilia (59, 60), monocyte dysfunction (59, 61), and ultimately profound lymphopenia through immune exhaustion (Fig. 2). IL-6 concentrations correlate with viral load and levels are highest prior to the need for mechanical ventilation (62). Early studies with tocilizumab, which binds soluble and membrane-bound IL-6 receptors, showed mixed results (63, 64). Subsequently the large REMAP-CAP and RECOVERY trials have shown a modest mortality benefit in a broad hospitalized population on concurrent glucocorticoid therapy (65, 66). Janus kinase inhibitors target key proinflammatory cytokines induced by COVID-19 (67). Baricitinib with remdesivir, but no glucocorticoid, has shown some efficacy at reducing recovery time, and tofacitinib with glucocorticoid reduced the composite outcome of death or respiratory failure (68, 69). Baricitinib may be an alternative to dexamethasone in some cases and is now being directly compared in ACTT-4 (70).

Trials targeting other inflammatory mediators are underway, although, surprisingly, the most abundantly used anti-inflammatory monoclonal antibodies, which target TNF and have been used in over 10 million autoimmune disease patients to date, have not yet been definitively evaluated for COVID-19 (71–74). IP-10 and MCP-1 may also be good candidate targets since their levels track with COVID-19 disease severity (75) and are associated with respiratory failure (76).

It is also surprising that methods to attenuate neutrophilia have not been investigated more thoroughly. Neutrophils in patients with COVID-19 display a range of abnormal features, including low density (59, 60), immaturity, a reduction in genes associated with degranulation (77), and enhanced production of highly inflammatory neutrophil extracellular traps (NET) (78). Colchicine, which targets neutrophils, has shown some preliminary efficacy restricted to early outpatient therapy (79), as in-depth immunological profiling shows that neutrophils far exceed any other immune cell type in the blood (61, 80) and lung (81) of COVID-19 patients. Similarly, monocytes, although an order-of-magnitude lower in number than neutrophils, also display abnormalities in the blood, with evidence of proliferation and reduced levels of cyclooxygenase 2 (59). The massive influx of activated neutrophils and monocytes in COVID-19 can also generate highly damaging reactive oxygen species in the lung. These species are unaffected by anticytokine therapy, but catalytic antioxidants, such as compounds of the Tris-malonic fullerene family, which are effective in multiple animal models, could be beneficial (82).

Timing of administration of anti-inflammatory therapy may impact efficacy. It is likely that early administration will result in the greatest benefit as it provides the best chance of preventing the cytokine storm and subsequent clinical deterioration. Current clinical trials and practice inadequately address this.

### Anticoagulation.

A striking difference between COVID-19 and other severe respiratory infections is the significantly higher incidence of pulmonary and extrapulmonary thromboses (83–86). It was initially thought that these thromboses were secondary to sepsis-associated disseminated intravascular coagulation. However, it is likely that excessive or dysregulated immuno-thrombosis is driving the mechanism for the hypercoagulable state, with myeloid cells cooperating with coagulation pathways (predominantly, platelets) to restrict pathogen spread (87–89).

The interaction between the immune system and coagulation pathways that leads to increased thrombosis is complicated. SARS-CoV-2 infects both the alveolar epithelium and endothelium via ACE2, facilitating extensive spread of infection, inflammation, and injury in alveolar-capillary beds (90). This inflammation and damage, coupled with increased platelet-vessel wall interaction, platelet activation, and reduced von Willebrand factor cleavage by reduced metalloprotease, are likely key contributors to thrombotic microangiopathy (91–93). Increased neutrophils, tissue macrophages, and abnormal monocytes and platelets (59) also contribute to abnormal immunothrombosis. NETosis in blood and tissue directly enhances thrombosis by triggering platelet activation and endothelial inflammation. Complement also contributes via C1q, C3, C3a, and C5a and the MAC complex to activate platelets and platelet-bound complement, which in turn increases the activity of neutrophils and NETosis (94).

In most countries, patients admitted to hospital are immediately started on anticoagulation therapy with low molecular weight or unfractionated heparin as thromboprophylaxis. The benefit of therapeutic levels of anticoagulation as thromboprophylaxis is less clear but is supported by early results from REMAP-CAP and ATTACC trials (95). Other nonheparin-based anticoagulants like rivaroxaban (factor Xa inhibitors) have been tested in the context of comparing therapeutic vs. prophylactic anticoagulation and do not improve clinical outcomes (96). Given the immune system-coagulation pathways interplay, the most effective therapy for prevention of thrombosis might be a combination of anticoagulants and anti-inflammatory drugs.

## Novel Therapeutic Approaches

### Inhaled Therapies.

Affordable treatments that are effective early in the disease course, easy to deliver, and have minimal side effects would greatly improve our response to the pandemic. Inhaled therapies could address this need, and many novel therapeutics could be delivered this way, including corticosteroids, IFN, aptamers, and fullerene-based antioxidants.

Inhaled corticosteroid (ICS) treatment may be effective in COVID-19 given that oral dexamethasone has shown benefit. ICS provides rapid delivery of corticosteroid directly to the lung, where it might down-regulate ACE2 expression and therefore SARS-CoV-2 entry (97). Early data have suggested the benefit of inhaled budesonide in early COVID-19 (98, 99). Well-established doses of inhaled corticosteroids have minimal systemic effects (100). Regulatory bodies in the United Kingdom have now endorsed ICS in nonhospitalized patients with the aim of preventing hospital admission.

Another inhaled option is IFN, a family of proteins that display profound antiviral activity. Studies of genetic susceptibility to severe infection and natural autoantibodies against IFN-α have revealed that the severity of COVID-19 is highly dependent on the endogenous level of type 1 IFN (101, 102). Inhaled delivery of IFN-β, which is not susceptible to antibody inhibition, has shown value in early-phase studies and is being assessed at a larger scale (103). The combination of ICS and IFN-β could target both the excessive host response and the virus early in the disease, reducing viral burden and hospitalization as well as transmission.

### Aptamers.

Aptamers are binding reagents made from modified nucleic acids, which have the same high affinity and specificity for pathogens as antibodies (104–106). A collection of modified DNA aptamers that target a broad range of epitopes on the SARS-CoV-2 spike protein have been generated and selectively bind to the S1 domain (including those that target the receptor-binding domain) or S2 domain. Aptamer-based reagents can also be made that can bind to the spike protein from both SARS-CoV and SARS-CoV-2 with comparable affinity. Some of these reagents potently inhibit the binding of the spike protein to its cell-surface receptor ACE2 and exhibit antiviral activity against SARS-CoV-2 virus, including many of the recent variants. Heterodimerization of modified aptamers that target nonoverlapping epitopes is a viable strategy for improving potency and reducing the impact of mutations on drug efficacy. Aptamers have several advantages for the treatment of COVID-19, including lower production costs than antibodies, no need for a cold chain, and the ability to be delivered by inhalation.

## Preparing Therapeutics for the Next Pandemic

The WHO International Health Regulations obliges countries to have “the capacity to respond promptly and effectively to public health risks and public health emergencies of international concern” (Article 13) (107). However, what this looks like is in the eye of the beholder and the perceived importance of pandemic preparedness will wane over time. There are numerous aspects to consider.

First, data from localized epidemics needs to be disseminated quickly, especially outbreaks of unknown diseases. The accelerated distribution of viral genomic sequences is critical to the development of diagnostics and therapeutics, especially vaccines.

There is a need to ensure existing patient datasets are ready to examine therapeutic hypotheses early in pandemics. OpenSAFELY, a platform that allows secure analysis of 24 million records in the UK National Health Service (7, 108, 109) provided insights into patient outcomes and the effect of concomitant medication use on COVID-19 outcomes, for example, nonsteroidal anti-inflammatory drug use and ICS. Similarly, large-scale global registries are equally important in providing information on preexisting medication use. Data from registries such as the COVID-19 Global Rheumatology Alliance and Surveillance Epidemiology of Coronavirus Under Research Exclusion (SECURE-IBD) helped to identify the ineffectiveness of hydroxychloroquine as a prophylactic therapy for infection with SARS-CoV2 (110) and the potential importance of therapies targeting TNF and IL-6 (111–115).

There is a need to prioritize vaccine and therapeutic research into agents with known pandemic potential, such as influenza, dengue, chikungunya, and hemorrhagic fever viruses like Ebola and Marburg. The burden from these agents disproportionately lies in low to middle income countries where surveillance and health infrastructure is least well-equipped to manage outbreaks.

Finally, creating a clinical trials system that is ready to deploy within days is critical to address important questions in therapeutics. The enthusiasm to fund extensive trial networks will undoubtedly wane in time, unless they either can address other important questions between pandemics, such as influenza, or only consume a very small resource to maintain their capacity between pandemics.

## Conclusion

This pandemic has put a strain on the global community that we have not seen outside wartime. In our interconnected world, we have seen that none of us are safe until we are all safe. Inevitably, the response has been incomplete, but in contrast to previous respiratory viral threats, we have seen progress in therapeutics and an extraordinary vaccine response.

Nevertheless, to keep pace with evolving unmet needs, drug development must adapt. Despite our improved knowledge of biology and inflammatory processes, most of the therapeutic approaches have been empiric. Future pandemic threats are inevitable and require a more focused response; we must translate biological insight into specific therapeutic hypotheses for testing. Thousands of small underpowered studies of myriad therapeutics have not delivered much insight. Targeted repurposing of existing therapeutics is vital in the early response to an emerging threat, but there needs to be an effective trial ecosystem to bring forward promising novel therapies.

We also must think beyond our current, limited field-of-view. Most of the current response has focused on preventing death in hospitalized patients. Effective treatments for earlier disease that can be deployed widely to prevent hospitalization have eluded us; oral and inhaled routes of delivery are important for mass deployment. These treatments are particularly important for less-wealthy countries in which hospital infrastructure is limited. Most conditions require combination therapy and we need to consider how the armamentarium can be used to maximal effect; this is likely to involve combining antiviral and immunomodulatory drugs. SARS-CoV-2 targeted antivirals are a clear gap, but host-directed antivirals and interventions based on specific cellular aspects of the inflammatory response may also be important. Host-directed antivirals may be particularly important because they are likely to have utility against many other viruses, and thus may prevent an epidemic turning into a pandemic.

Pandemics require a rapid coordinated response (Box 1). Maintaining extensive research and development infrastructure on a “just in case” basis is prohibitively expensive and impractical. Pivoting existing frameworks addressing endemic disease between pandemics is a more practical approach. Although the global response to COVID-19 has been highly variable, there are important examples of success that we must use to shape our response for the future. To provide the readiness the world needs and deserves, we must learn from the past but think differently in the future.

Box 1. **Important messages for the future**

• We have seen improvements in hospitalized patient care, especially use of dexamethasone. Great unmet needs are treatments to keep patients out of the hospital.
• Timing of therapeutic delivery is critically important, with lack of efficacy and even deleterious effects if given at the wrong time.
• Cost-effective drugs are essential, with oral or local delivery. Local delivery of steroids, anti-inflammatories, and antivirals by inhaler/nebulizer needs to be optimized.
• Only large-scale phase 3 trials can deliver quality of data to change medical practice, not underpowered small trials.
• New drugs and drug classes need to be delivered: for example, direct-acting antivirals for COVID-19 and host-targeting antivirals that work independent of viral mutations.
• Development of oral broad-spectrum host-targeting antivirals should be a priority for preventing future viral epidemics from turning into pandemics.

## Data Availability

All study data are included in the main text.
